# South American H4N2 influenza A virus improved replication in chicken trachea after low number of passages

**DOI:** 10.3389/fvets.2023.1182550

**Published:** 2023-05-31

**Authors:** Lucas M. Ferreri, Silvia Carnaccini, Valeria Olivera, Ariel Pereda, Daniela Rajao, Daniel R. Perez

**Affiliations:** ^1^Department of Population Health, Poultry Diagnostic and Research Center, College of Veterinary Medicine, University of Georgia, Athens, GA, United States; ^2^Instituto de Virologia CICVyA, Instituto Nacional de Technologia Agropecuaria (INTA), Castelar, Buenos Aires, Argentina; ^3^Programa Nacional de Sanidad Animal, Instituto Nacional de Technología Agropecuária (INTA), Castelar, Buenos Aires, Argentina

**Keywords:** influenza A virus, adaptation, subtype H4N2, poultry, waterfowl

## Abstract

Introduction of influenza A viruses (FLUAV) into poultry from waterfowl is frequent, producing economic burden and increasing the probability of human infections. We have previously described the presence of FLUAV in wild birds in Argentina with unique evolutionary trajectories belonging to a South American lineage different from the North American and Eurasian lineages. Adaptability of this South American lineage FLUAV to poultry species is still poorly understood. In the present report, we evaluated the capacity of an H4N2 FLUAV from the South American lineage to adapt to chickens after low number of passages. We found that five mutations were acquired after five passages in 3-days-old chickens. These mutations produced a virus with better infectivity in *ex vivo* trachea explants but overall lower infection in lung explants. Infection of 3-week-old chickens persisted for a longer period and was detected in more tissues than the parental virus, suggesting adaptation of the H4N2 influenza A virus to chicken.

## Introduction

Influenza A viruses (FLUAV) from waterfowl are of global concern. Multiple pandemics have occurred as the product of genetic exchange between FLUAV circulating in humans and the avian reservoir (1). FLUAV contains eight gene segments, two of which encode for the surface glycoproteins hemagglutinin and neuraminidase (HA and NA, respectively). The other six for the internal gene cassette, comprised by the set of polymerases, PB2, PB1 and PA, the nucleoprotein NP, the matrix segment M and NS, which encodes for nonstructural proteins. While HA and NA are responsible for the entrance to and exit from the cell, the internal genes perform replication, innate immune evasion, and physical support among other functions. The 18 HA and 11 NA subtypes of FLUAV are classified by their antigenic properties whereas the internal gene cassettes are divided by their origin. Three major lineages of avian FLAUV internal genes have been described: Eurasian, North American, and South American lineages (2).

Among the most prevalent FLUAV circulating in waterfowls, the H4 subtype has been detected frequently in domestic avian species across Asia, Europe, and North America since it was first isolated from a duck in 1956, highlighting its wide distribution (3). Furthermore, some H4s have been shown to infect mice (4–6), pigs (7), and marine mammals (8) without prior adaptation, indicating ability to switch hosts between avian and mammalian species.

Chicken (*Gallus gallus domesticus*) meat and eggs are among the most consumed animal-based proteins, making this species widely distributed worldwide. Chickens are risen in large numbers and found at high densities within commercial farms, a scenario that increases the probability of virus transmission and adaptation upon new introductions. Chickens can be infected with a broad range of FLUAV and can act as intermediate species between waterfowl and mammalian hosts (9, 10). Studies showed that farmers, workers, and veterinarians in the poultry industry are at higher risk of infection with zoonotic FLUAVs, including H4 viruses (11, 12).

In the present work, we evaluated the adaptability of a H4N2 influenza A virus, from the South American lineage isolated from silver teal (*Anas versicolor*) in Argentina. We found that mutations were acquired after five passages, and that, even though absent or at low frequency in nature, they conferred better replication *in vivo*. Furthermore, we show fitness improvement in the trachea but not in the lungs, suggesting that the acquired substitutions were positively selected. Overall, we show that the H4 subtype from the South American lineage isolated from wild birds requires only a few passages to adapt to chicken.

## Materials and methods

### Viruses and cells

The isolation of the duck A/silver teal/Argentina/CIP051-32/2011 (H4N2) virus (32/H4N2) has been previously described (2). The P5Ch32/H4N2 virus was sequenced from lung and trachea homogenates after five passages of 32/H4N2 in 3-days-old chickens. Viruses 32/H4N2 and P5Ch32/H4N2 were produced by reverse genetics as previously described (13) and stocks were generated in 10-day-old embryonated chicken eggs. Madin-Darby canine kidney cells (MDCK cells) were used for either titration by tissue culture infectious dose 50 (TCID_50_) or plaque assay. Read out for TCID_50_ was performed by Reed and Muench as previously described (14).

### Chickens and housing

Specific-pathogen-free (SPF) White Leghorn (*Gallus gallus domesticus*) embryonated eggs (Rosenbusch S.A. CABA, Argentina) were purchased, incubated, and hatched in an automatic incubator (Yonar, CABA, Argentina). Groups were housed separately in sterilized isolators for chickens under negative pressure conditions (Allentown CH8ISOL) with food and water *ad libitum* throughout the experiments. Animal care was performed in accordance with the approved protocols of the National Institute of Agricultural Technology Ethics Committee (INTA, Argentina; protocols numbers 26/2013 and 44/2014).

### Serial lung passages in 3-days-old chicken

Serial lung passages started with the infection of three 3-days-old chicken inoculated intratracheally with 1 × 10^6 TCID_50_/bird of allantoid fluid in PBS. At 3 dpi, the chickens were sacrificed, whole lungs were collected; lung homogenates were prepared with a mortar and sterile sea sand. The pool of lung homogenates from passage 1 was used to infected animals in passage 2. From passage 2 to 4, the lung homogenate with the lowest Ct value was used to inoculate the next group of chickens. The homogenates were resuspended in 5 mL of PBS with antibiotics penicillin 10,000 IU/mL, streptomycin 5 mg/mL, gentamicin Sulfate 1 mg/mL, kanamycin sulfate 700 μg/mL and amphotericin B 10 μg/mL (Sigma Chemical Co, St. Louis, MO, United States), clarified by centrifugation at 1,500 rpm for 20 min at 4°C and store at – 80°C until used. Inoculum for serial passages were obtained using 100 μL of clarified lung homogenates from the bird with lowest Ct values. The number of birds per group varied from 3 to 5 based on availability of SPF embryonated eggs at the time of the experiments. Birds were observed daily for clinical signs of infection and general well-being. Experiments were carried out under BSL-3 conditions with investigators wearing appropriate protective equipment and compliant with animal care approved protocols of the National Institute of Agricultural Technology Ethics Committee (INTA, Argentina; protocols numbers 26/2013 and 44/2014).

### Infection of 3-weeks old chicken

To assess infectivity of the 32/H4N2 virus, two experiments of four 3-week-old chickens were performed. Chickens were inoculated with 1 × 10 ^ 6 TCID_50_/bird of allantoid fluid diluted in PBS. Sampling was performed by swabbing trachea and cloaca at 2, 3 and 4 dpi. Two chickens were euthanized at 3 and 4 dpi for lung tissue collection. Lung homogenates for virus detection were prepared as described above. For inoculation with the P5Ch32/H4N2 virus, we performed one experiment with eight chickens. Chickens were also inoculated with 1 × 10 ^ 6 TCID_50_/bird of allantoid fluid diluted in PBS. Sampling was performed by swabbing trachea and cloaca at 2, 3 and 4 dpi. Four chickens were euthanized at 3 and 4 dpi for lung tissue collection. Lung homogenates for virus detection were prepared as described above.

### Detection by qRT-PCR

The viral RNA was extracted from 140 μL of either lung homogenates or tracheal swabs using a QIAamp Viral RNA Mini Kit (Qiagen Inc.). Viral cDNA was prepared with random hexamers using a High Capacity cDNA Archive kit (Applied Biosystems, Foster City, CA, United States). FLUAV detection was done by real-time reverse transcriptase PCR (RT-qPCR) using TaqMan Universal PCR Master Mix (Applied Biosystems) directed to the matrix (M) gene described elsewhere (15). The PCR reaction was performed on an ABI Prism 7,500 SDS (Applied Biosystems). Samples with a CT value of less than or equals to 38 were considered positives.

### Sequencing

Total RNA was extracted using the QIAamp viral RNA, Mini kit (Qiagen, Valencia, CA, United States) according to manufacturer’s instructions. Reverse transcription was carried out with the uni-12 primer (5′-AGCAAAAGCAAAGG-3′) and AMV reverse transcriptase (Promega, Madison, WI, United States). PCR amplification was performed using specific primers. The PCR products were sequenced using BigDye-Terminator protocol V3.1 (Applied Biosystems, Foster City, CA, United States).

### Plaque assay and plaque size measurement

Six-well plates with confluent Madin-Darby canine kidney cells were infected with either 32/H4N2 or P5Ch32/H4N2 in PBS. After 1 h of adsorption, the monolayer was washed twice and overlay with agar media (2 × MEM media, Gibco, ThermoFisher Scientific, Waltham, MA, United States), L-glutamine (4 mM; VWR, Radnor, PA, United States), sodium bicarbonate (0.3%; VWR), BSA (0.5%; Sigma), HEPES (30 mM; Lonza, Basel, Switzerland), 0.8% agar (Oxoid, ThermoFisher Scientific), 1% DEAE dextran [ThermoFisher Scientific, 1 μg/ml TPCK-treated trypsin (Sigma)]. After 48 h, cells were fixed with 4% formaldehyde and stained with crystal violet (Sigma). Size of plaques were manually measure using Fiji ImageJ2 version 2.9.0/1.53 t.

### Lung and trachea explants

Lungs and tracheas were used for explant studies (16, 17). Tissues were obtained from 3-weeks old SPF White Leghorn chickens. Using aseptic technique, the tracheas were dissected from the animal, and washed with wash media (PBS containing Penicillin (200 U/mL), Streptomycin (200 μg/mL), Amphotericin B (25 μg/mL)). Tracheal rings of equal width (0.5 cm of length) were infected with 10 ^ 4 TCID_50_/mL of virus for 1 h at 37°C and 5% CO2. Afterwards, explants were washed 5 × in wash media. Trachea explants were placed on an air-liquid interface supported by a gauze pedestal of approximately 1 cm^2^ embedded in Trachea explant media 250 mL DMEM/F12, no phenol, 250 mL RPMI, no phenol, Penicillin (200 U/mL), Streptomycin (200 μg/mL), Amphotericin B (0.1 μg/mL), Gentamicin (0.2 μg/mL), Glutamine (0.3 ng/mL), 5 mL 100 × amino acids (ThermoFisher Scientific) in 12-well plates (ThermoFisher Scientific), and incubated for 72 h at 37°C and 5% CO2.

For lung explants, the desired lobes were dissected from the birds and washed 5 × with Lung explant media (500 mL M199, no phenol), Penicillin (200 U/mL), Streptomycin (200 μg/mL), Amphotericin B (0.1 μg/mL), Gentamicin (0.2 μg/mL), 5 mL Vitamin supplement (ATCC), 5 mL 100 × amino acids (ThermoFisher Scientific), 5 mL ITS 100 × (Insulin, Transferrin, Selenium; ThermoFisher Scientific), Hydrocortisone (0.5 μg/ml). Pieces of lungs were obtained using 4 mm biopsy punches (VWR). Infection and cultivation of lung explants was done as with trachea explants. Collection of supernatants from kinetic growth experiments in both types of explants was done adding 200 μL of tissue-specific media before collecting 200 μL of media containing the virus. Titration was performed by TCID_50_ as described above.

### Statistical methods

Plaque size comparison between the 32/H4N2 virus and the P5Ch32/H4N2 virus was done by two sample unpaired t-test. For evaluating significance for growth kinetics in explants, Welch two sample t-test was used. All tests were performed in rstatix (version 0.7.2), from R version 4.1.3.

## Results

### Low fitness of 32/H4N2 in 3-weeks-old chickens

We evaluated infection capacity of the duck isolate A/silver teal/Argentina/CIP051-32/2011(H4N2; 32/H4N2) in 3-weeks-old chickens (*Gallus gallus domesticus*). Two groups of four chickens produced infection detectable only at 2 days post inoculation (dpi; Table 1).

**Table 1:** The P5Ch32/H4N2 virus is detected for longer and in more tissues in 3-weeks old chicken than the parental 32/H4N2 virus.

Tissue	Virus	Days post infection		
		2	3	4
Trachea	32/H4N2	33.1 (4) 31.5 (4)	*bld* (4/4)	*bld* (2/2)
	P5Ch32/H4N2	31.75 (8)	28.78 (8)	26.67 (4)
Cloaca	32/H4N2	*bld* (4/4)	*bld* (4/4)	*bld* (2/2)
	P5Ch32/H4N2	*bld* (8)	28.34 (8)	31.2 (4)
Lung	32/H4N2	*na*	*bld* (2/2)	*bld* (2/2)
	P5Ch32/H4N2	*na*	30.35 (4)	*bld* (4)

Values for the infection with the 32/H4N2 virus represent two experiments of four chickens whereas values for the infection with the P5Ch32/H4N2 virus represent one experiment of eight chickens. Numbers show mean Ct values from samples whereas values between brackets show number of birds from which the mean Ct values were calculated. Trachea and cloaca were sampled by swabbing whereas lungs represent homogenates. “*bld*” = below limit of detection. “*na*” = not assessed.

### Low number of passages of the 32/H4N2 virus in chickens resulted in mutations with differential fitness

**Figure 1:**
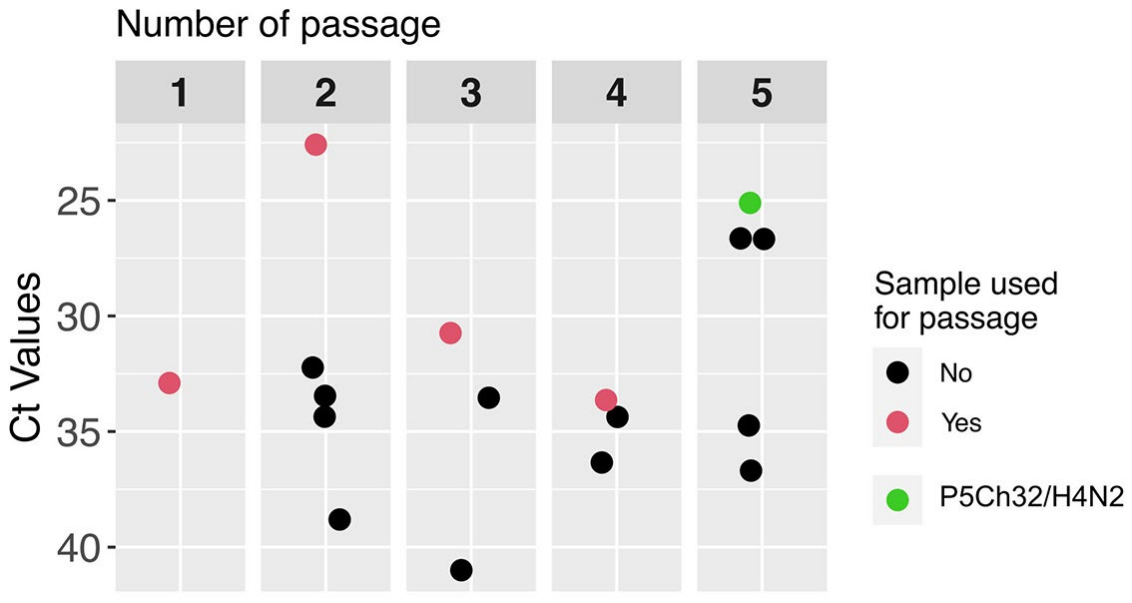
Passages of A/silver teal/Argentina/CIP051-32/2011(H4N2; 32/H4N2) in 3-days old chickens. Adaptation was attempted by serially passaging the 32/H4N2 virus in specified pathogen-free chickens. In passage 1, the pool of lung homogenates were utilized as inoculum for passage 2. Afterwards, the lung homogenate with the lowest Ct value within the group (red dots) was utilized as inoculum for the next group. The green dot represents the chicken from which the P5Ch32/H4N2 virus was characterized.

To evaluate the adaptability of the 32/H4N2 virus to chicken, we performed five passages of lung homogenates in 3-days-old chickens (18). Initially, three chickens were inoculated with 1 × 10 ^ 6 TCID_50_. Lung homogenates from the bird with the lowest Ct value were used to perform the serial passages (Figure 1). No overt clinical signs were observed. We characterized phenotypically and genetically the virus from the bird with the lowest Ct value in the passage 5 group (P5Ch32/H4N2 virus).

**Figure 2:**
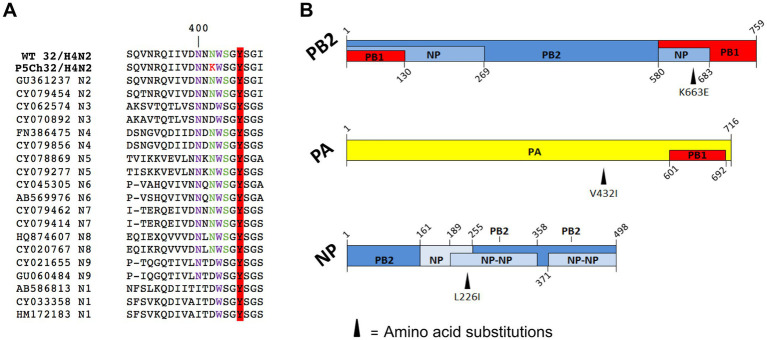
Amino acid substitutions present in P5Ch32/H4N2. **(A)** Substitution NA N402K (N2 numbering; in red) acquired after passaging in chickens disrupts a putative N-glycosylation site (green). From the canonical sialic acid recognition site, the tyrosine at position 406 is highlighted in red. In purple, sites corresponding to the secondary binding site (19). **(B)** Amino acid substitutions in proteins involved in the ribonucleoprotein complex. This figure is an adaptation from Boulo et al. and Naffakh et al. (20, 21).

**Figure 3:**
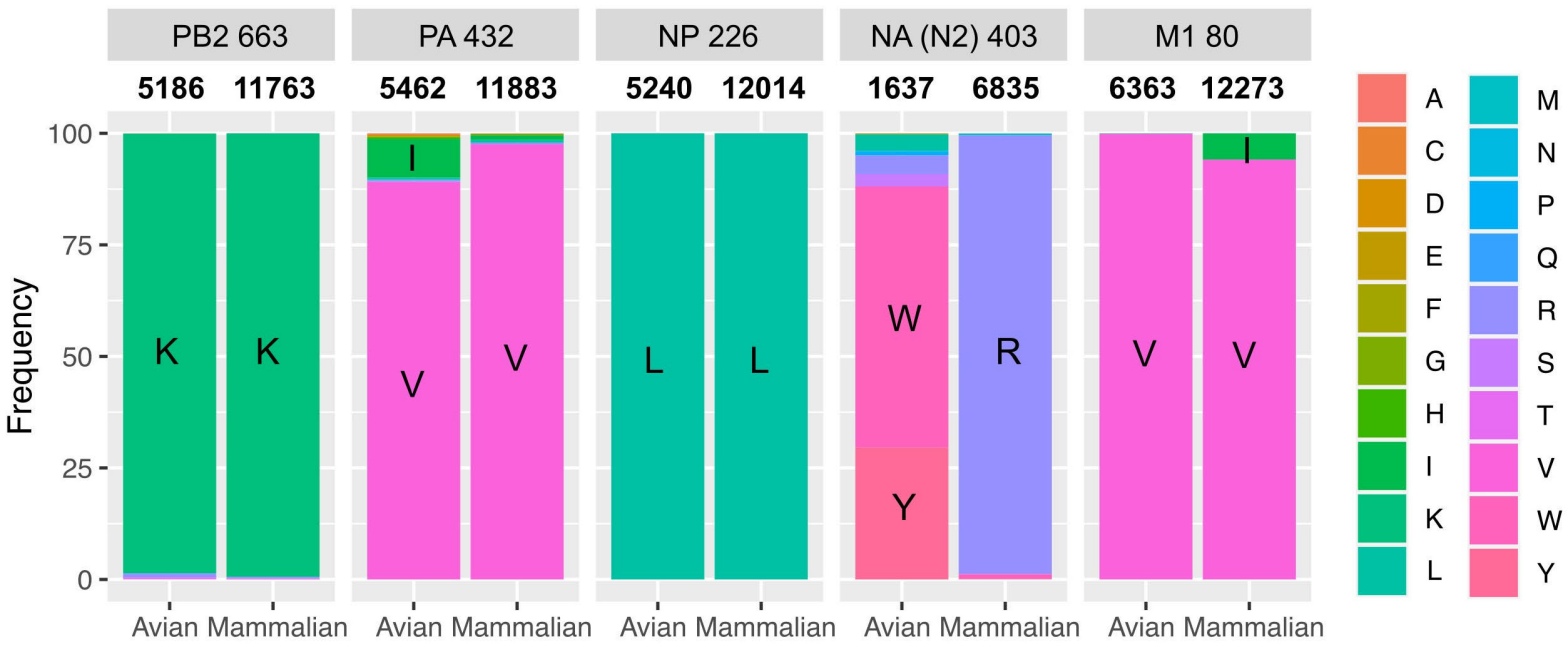
Absence of molecular markers from P5Ch32/H4N2 suggest fitness cost in nature. NCBI database was searched for NA N403K, PB2 K663E, PA V432I, NP L226I and, M1 V80I. Numbers between title of the panel and bar plots show the total number of sequences analyzed.

Sequencing the P5Ch32/H4N2 virus from lung and trachea homogenates revealed the same amino acid substitutions in both organs: NA N402K, PB2 K663E, PA V432I, NP L226I, and M1 V80I (Figure 2). We then evaluated the presence of these molecular markers in nature, which would help understanding their fitness in different hosts. We evaluated internal genes from all subtypes and N2 NA regardless of its HA subtype association. The PA V432I mutation was present at ~ 8% of FLUAV sequences detected in avian hosts and at 1% in mammalian hosts. The NA N402K mutation was detected at ~ 0.05% of N2 FLUAV sequences detected in mammalian hosts and the M1 V80I mutation was present at > 5% of FLUAV sequences in mammalian species (Figure 3).

**Figure 4:**
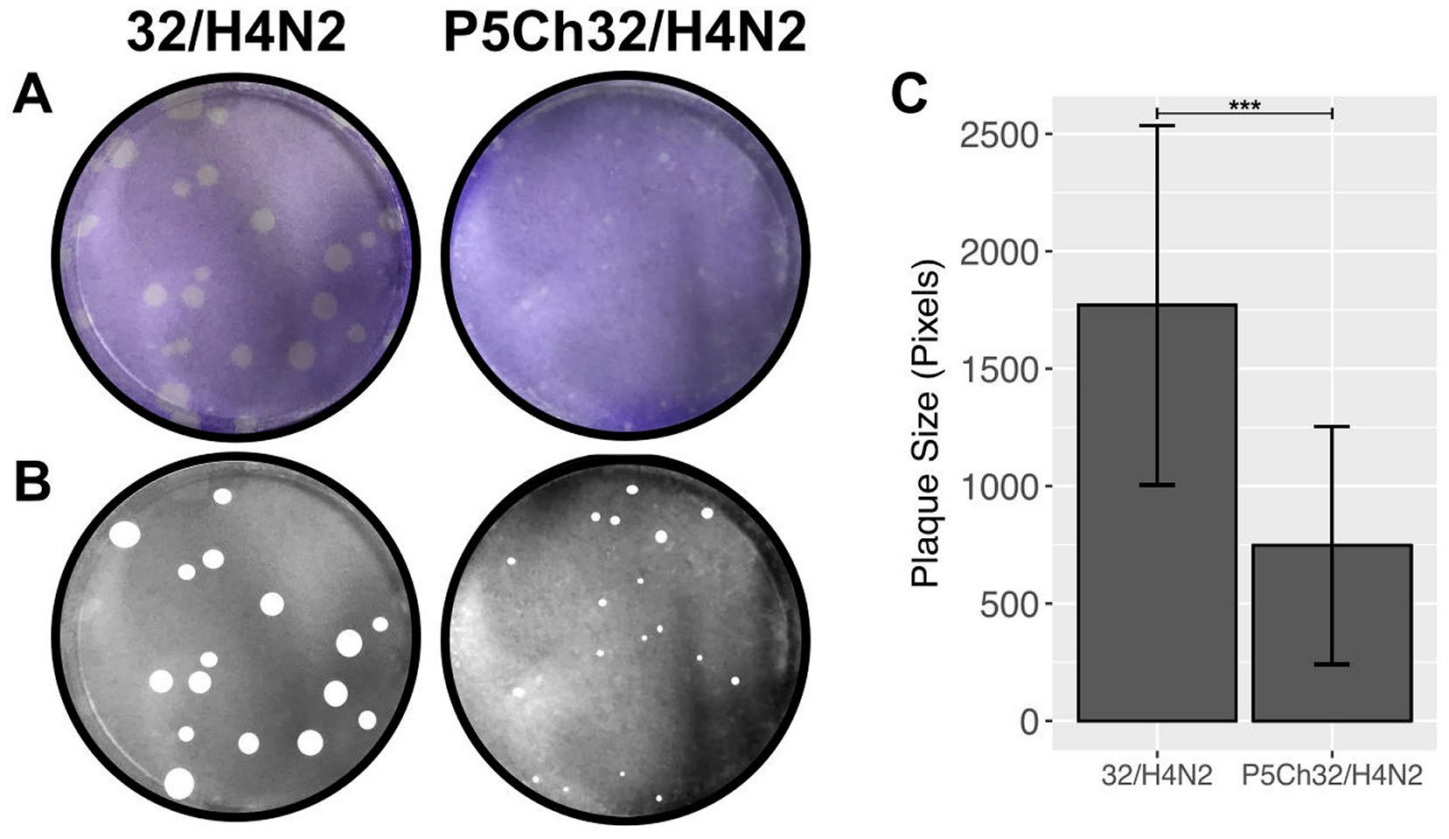
*In vitro* fitness differences conferred by mutations in P5Ch32/H4N2. Plaque size is indicative of fitness differences *in vitro*. Plaque formation from infection in Madin-Darby canine cells was evaluated after crystal violet staining **(A)**. Plaques were manually measured **(B)** and total area of plaques was evaluated **(C)**. Plaque size was statistically significant (^***^p = 0.000106, two samples unpaired *t*-test). Bars represent the mean size of the plaques and bars, the standard deviation.

The size of a plaque produced by cytopathic effect is a direct measurement of fitness *in vitro*. We compared the size of plaques to evaluate whether the mutations acquired after passages in young chickens resulted in fitness differences (Figure 4). While the wild type 32/H4N2 virus produced large plaques, the P5Ch32/H4N2 virus produced pinpoint plaques, demonstrating that the mutations associated with the P5Ch32/H4N2 virus had impacted its fitness *in vitro*.

### The P5Ch32/H4N2 virus improved fitness in trachea

**Figure 5:**
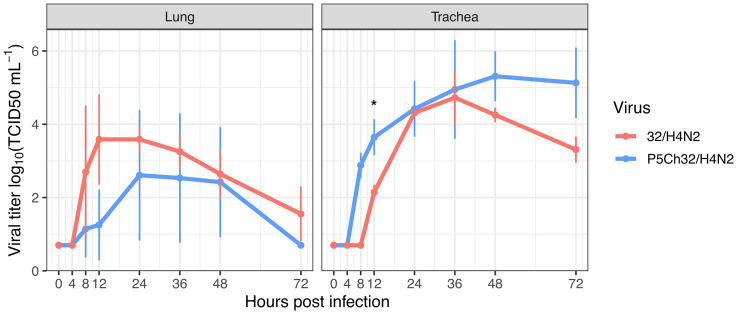
Trend of higher replication in chicken trachea upon infection with P5Ch32/H4N2 virus. While in lung explants the P5Ch32/H4N2 virus seemed to have a cost early in the infection at 12 h post infection (No statistical significance), passages conferred better replication in trachea with statistical significance at 12 hpi (^*^p = 0.02082; Welch two sample *t*-test). Data represents two independent experiments with three replicates each. Dots represent the mean titer and bars represent their standard deviations.

We infected trachea and lung explants, to evaluate whether the mutations present in the P5Ch32/H4N2 virus confer differential replication in these tissues. For the P5Ch32/H4N2 virus, replication in the lung decreased compared to the 32/H4N2 virus, but the differences did not reach statistical significance (Figure 5). In the trachea, overall replication was improved for the P5Ch32/H4N2 virus, reaching statistical significance at 12 h post-infection (hpi). Even though between 24 and 36 hpi both viruses replicated at similar titers, at 48 and 72 hpi the P5Ch32/H4N2 virus maintained higher titers than the 32/H4N2 virus. Overall, these data suggest that the acquisition of the mutations after the passages conferred a replicative improvement in the trachea.

### The P5Ch32/H4N2 virus improved fitness *In vivo*

Since the 32/H4N2 virus showed to be deficient to replicate in 3-week-old chickens, we assessed the capacity of the P5Ch32/H4N2 virus to produce infection in chickens of the same age. The group of birds infected with the P5Ch32/H4N2 virus tested positive for virus shedding until the last day evaluated, 4 dpi. Furthermore, virus was also detected in cloacal swabs at 3 and 4 dpi as well as in lung homogenates at 3 dpi (Table 1; Supplementary Table 1). Overall, these data showed that the P5Ch32/H4N2 virus had improved shedding and extended tissue tropism compared to its ancestral 32/H4N2 virus.

## Discussion

Introduction and adaptation of viruses from their natural hosts are key for the establishment of novel viruses into new species. Wild waterfowls, from the order Anseriformes and Charadriiforms, are the natural host for FLUAV. Close contact of poultry with species from these two orders has been reported (22), helping disseminate FLUAV to domestic avian species and the consequent host switch for low and high pathogenicity FLUAV. In the present work, we aimed to evaluate the adaptation of a H4N2 avian influenza virus from the South American lineage isolated from a wild bird. After five passages in young chickens, we found that the mutations acquired conferred a replicative advantage in 3-weeks-old chicken and in trachea explants suggesting that the passages promoted selection for infection in chicken.

The South American lineage represents a unique evolutionary clade with little information about its adaptability (2, 23, 24). Rimondi et al. (23) recently showed that 20 passages in 3-weeks old chickens produced 13 amino acid substitutions that conferred a South American H6N2 capacity to increase replication in chickens. Here, we found that five amino acid substitutions arose after only five passages in 3-days old chickens. This experimental setup allowed us to make the process of adaptation more efficient because young chickens do not have their innate immunity fully developed, lowering innate immune pressure (25). Moreover, a high number of passages increases the probability of mutations being fixed by chance interfering with the process of positive selection.

The mutations acquired after passages were present in NA, PB2, PA, NP, and M1 proteins. Even though at low frequency, NA N402K, PA V432I, and M1 V80I were found in viruses isolated from mammals suggesting that these viruses are capable of infecting species other than avian hosts. Conversely, the PA V432I mutation was found in ~ 8% of the avian and 1% in mammalian FLUAV sequences deposited in public databases. This reflects the capacity of viruses with the PA V432I substitution to infect mammals and birds.

The fact that 3 out of 5 mutations are present in the ribonucleoproteins is indicative of a process of adaptation that involves the replicative machinery of the virus. The mutation PB2 K663E falls in the 627 domain—a distinctive domain protruding from the polymerase core. The domain 627 was previously shown to play a role in the adaptation of avian viruses to mammals (26). Differences in the biology of infection from waterfowl to chicken is likely one of the main host restrictions since FLUAV replicate in the gastrointestinal tract in waterfowl whereas is mainly a respiratory infection in poultry. Therefore, the mutations found in the proteins involved in the formation of the ribonucleoproteins may moderate efficiency of replication under these two different environments.

Another salient mutation is NA N402K. This mutation falls in a region of the protein that has the potential to disrupt a putative glycosylation site, and/or to modify the secondary binding site (19). Furthermore, site 406Y, which forms part of the primary sialic acid recognition site, is adjacent. This can have implications in the performance of the NA and the sialic acid species it recognizes. It has been shown that the epithelial cells from the respiratory tract of chickens contain both sialic acid in α2,3 and α2,6 conformation whereas the α2,3 conformation is predominant in the gastrointestinal tract of waterfowl (9, 27–29). Therefore, mutations in either of the proteins involved in the recognition of these ligands are expected to change upon host switch. Which of these mutations are the ones responsible for the infection improvement in 3-weeks-old chickens and whether they confer transmission capacity warrants further research.

It has been shown that selective pressure acts differently across various anatomical sites within the infected individuals (30). In our experiment of adaptation, we utilized lung homogenates to make serial passages but, in the *ex vivo* infection, fitness gain was only detected in the trachea. This can be attributed to the use of intratracheal inoculation during the passages. We argue that even though viruses were homogenized and collected from the lungs, intratracheal inoculation allowed the virus populations produced in the lungs to be selected in the trachea. Furthermore, the P5Ch32/H4N2 virus was mainly detected in the trachea of infected chicken and its robust infection in the trachea explants further support the notion that the trachea acted as a compartment where selection occurred, creating fit populations therein (31, 32).

Poultry species have been proposed as intermediate hosts between waterfowl and humans (9, 10). Even though human infections by H4 subtypes of FLUAV are still limited, the fact that FLUAV evolve rapidly in poultry (33) in addition to the close contact of the human population with these birds, sets the conditions for spillovers, increasing the probability of the establishment of new viruses in the human population.

## Data availability statement

Nucleotide genomic sequences from all Guatemalan viruses have been deposited at the NCBI Database under the following accession numbers OQ821651–OQ821658.

## Ethics statement

The animal study was reviewed and approved by National Institute of Agricultural Technology Ethics Committee (INTA, Argentina) (protocols numbers 26/2013 and 44/2014).

## Author contributions

LMF conception of the work, performed experiments, analyzed data, and wrote and edited the manuscript. SC and VO performed experiments. AP conception of the work and edited the manuscript. DR edited manuscript. DRP conception of the work, wrote and edited the manuscript. All authors contributed to the article and approved the submitted version.

## Funding

This research was funded by a subcontract from the Center for Research on Influenza Pathogenesis (CRIP) to DRP under contract HHSN272201400008C from the National Institute of Allergy and Infectious Diseases (NIAID) Centers for Influenza Research and Surveillance (CEIRS). This research was also funded by the Instituto Nacional de Tecnología Agropecuaria (INTA; PNSA 1115052 and PNSA 1115056) to AP. This work was also supported by CONICET (Consejo Nacional de Investigación Científica y Técnicas) and CIC (Comisión de Investigaciones Científicas), through fellowships given to ML.

## Conflict of interest

The authors declare that the research was conducted in the absence of any commercial or financial relationships that could be construed as a potential conflict of interest.

## Publisher’s note

All claims expressed in this article are solely those of the authors and do not necessarily represent those of their affiliated organizations, or those of the publisher, the editors and the reviewers. Any product that may be evaluated in this article, or claim that may be made by its manufacturer, is not guaranteed or endorsed by the publisher.
