# Supplementary material for: South American H4N2 influenza A virus improved replication in chicken trachea after low number of passages
Source: Front Vet Sci. 2023 May 31;10:1182550. doi: 10.3389/fvets.2023.1182550 (PMC10264679; doi:10.3389/fvets.2023.1182550)
Supplement: Supplementary file 1 [file Table_1.docx]

**Supplementary table 1. Infection with P5Ch32/H4N2.** “bld” = below limit of detection (>38 cycles). “na” = not assessed.

| **Virus** | **Sample** | **Chicken ID** | **2 dpi** | **3 dpi** | **4 dpi** |
| --- | --- | --- | --- | --- | --- |
| P5Ch32/H4N2 | Tracheal Swab | 1 | 37.15 | 34.16 | *na* |
|  |  | 2 | 29.75 | 27.91 | *na* |
|  |  | 3 | 33.58 | 29.87 | *na* |
|  |  | 4 | 30.45 | 29.13 | *na* |
|  |  | 5 | 27.3 | 25.68 | 28.98 |
|  |  | 6 | 27.4 | 27.99 | 21.79 |
|  |  | 7 | 36.53 | 28.75 | 32.65 |
|  |  | 8 | 31.9 | 26.79 | 23.28 |
|  | Cloaca | 1 | *bld* | *bld* | *na* |
|  |  | 2 | *bld* | *bld* | *na* |
|  |  | 3 | *bld* | *bld* | *na* |
|  |  | 4 | *bld* | 30.27 | *na* |
|  |  | 5 | *bld* | 26.39 | 32.59 |
|  |  | 6 | *bld* | 28.3 | 28.5 |
|  |  | 7 | *bld* | *bld* | 30.27 |
|  |  | 8 | *bld* | 28.39 | 33.4 |
|  | Lung | 1 | *na* | 32.45 | *na* |
|  |  | 2 | *na* | 29.1 | *na* |
|  |  | 3 | *na* | 30.8 | *na* |
|  |  | 4 | *na* | 29.05 | *na* |
|  |  | 5 | *na* | *na* | *bld* |
|  |  | 6 | *na* | *na* | *bld* |
|  |  | 7 | *na* | *na* | *bld* |
|  |  | 8 | *na* | *na* | *bld* |
